# ‘Cleaning up’ the neighbourhood: Affective dynamics of environmental gentrification

**DOI:** 10.1177/02637758251387040

**Published:** 2025-10-21

**Authors:** Loren March

**Affiliations:** Department of Geography and Planning, University of Toronto, Toronto, Canada

**Keywords:** Affect, environmental gentrification, fear, toxicity

## Abstract

This article examines how affect shapes trajectories of environmental gentrification through a study of greening in several downtown neighbourhoods in Toronto, Canada. Following the 1990s, two decades of greening practices in the West End neighbourhoods of Wallace-Emerson, Bloorcourt, Bloordale, and Sterling Junction have produced geographies of exclusion wherein unruly ‘toxic’ beings have been vilified, policed, and targeted for removal. An affective account of gentrification reveals how certain bodies become marked for intentional displacement. The author explores fear as a powerful affective force, used to justify various environmental and affective practices directed towards ‘cleaning up’ the neighbourhood. These practices serve to disrupt existing more-than-human relations, displacing and marginalizing inhabitants who are understood as ‘invasive’ outsiders and as part of an assemblage of waste that must be removed. Drawing upon queer scholarship and theory, this article delves into the place-specific intertwining of environmental remediation or ‘cleanup’ with social cleansing, arguing that invasiveness, toxicity, and non-belonging are affectively produced.

## Introduction

Exclusionary dynamics in cities are shaped by affect and emotion. This article explores how these forces drive and justify environmental gentrification, examining affective dynamics that have shaped post-industrial urban ecologies in parts of Toronto's West End. I focus on affective atmospheres of anxiety and fear that emerged between the 1980s and early 2000s, and on affective relations involved in efforts to ‘clean up’ certain stigmatized West End neighbourhoods. These affective dynamics serve to ‘queer’ more-than-human urban ecologies, constructing marginalized bodies as invasive outsiders, with harmful effects. Environmental cleanup and social cleansing dovetail, as assemblages of bodies in the study area became saturated with feelings such as suspicion, anxiety, and fear, justifying their intentional elimination.

Critical theories of gentrification emphasize the direct or indirect displacement of marginalized, poor, and low-income residents as a key consequence of the reinvestment of capital into parts of the city (see Lees, Slater and Wyly, 2008; Marcuse, 1985; Slater, 2006, 2009; Smith, 1996). Gentrification involves both residential or physical displacement, and ‘affective displacement’ (Butcher and Dickens, 2016), wherein some residents manage to remain in place as gentrification proceeds but experience the disintegration of a place and subsequent exclusion as something jarring that involves intense feelings of disorientation, loss, grief, and resentment. Affect, as such, is revealed to be political. Investigations of affective dynamics in gentrification offer nuanced insights into how this process takes place, thus being complementary to other critical approaches. This article attunes to the role of affect in driving various forms of displacement, showing how displacement is not only concurrent with gentrification, but may also be targeted and intentional.

I examine environmental gentrification with an attention to both affect and more-than-human worlds, in response to calls to attend to non-human beings and politics (Hubbard and Brooks, 2021; Kocisky, 2021; March and Bunce, 2022), as well as the emotional dynamics that play out in gentrification (Seitz and Proudfoot, 2021). Such attention adds complexity to our storyings of gentrification, helping us to understand its workings, its everyday worlds, and how it is experienced. I explore emotional factors underpinning how greening becomes a ‘grammar of moral action’ (Angelo, 2021: 5), expanding on how that which we understand as ‘green’ – from greening practices to green consumer products, amenities, and non-human beings categorized as such – becomes a ‘good’ solution to urban problems through the workings of an affectively informed imaginary. I also show how toxicity affectively comes together, marking certain marginalized bodies as out of place and needing to be removed. In this, I expand upon existing theorizations of toxicity in environmental gentrification scholarship (for example, Anguelovski, 2016; Dillon, 2014; Kern, 2015), showing how it takes shape and has effect in place- and context-specific ways.

The place I focus upon includes several neighbourhoods in west downtown Toronto, including Wallace-Emerson, Bloorcourt, Bloordale, and Sterling Junction. It stretches over 3.5 km from east to west (see Figure 1). This article focuses mainly on the western end of this study area, a section that was the focus of media attention through the 1990s and 2000s, was stigmatized, and was targeted for an array of planning, policy, and policing interventions. This area has historically been home to diverse populations, including large Portuguese and Italian communities. Working-class and immigrant families have long settled in the western end of the study area, drawn by its relatively affordable housing stock and proximity to industrial employment. Through the 1980s and 1990s, these neighbourhoods also welcomed growing Latin American, Caribbean, and later South Asian and African immigrant groups, reflected in the changing mix of businesses, community infrastructure, and services. Census data from this period shows higher proportions of renters than the city average, a concentration of low- to moderate-income households, underscoring the area's working-class character.

**Figure 1.**
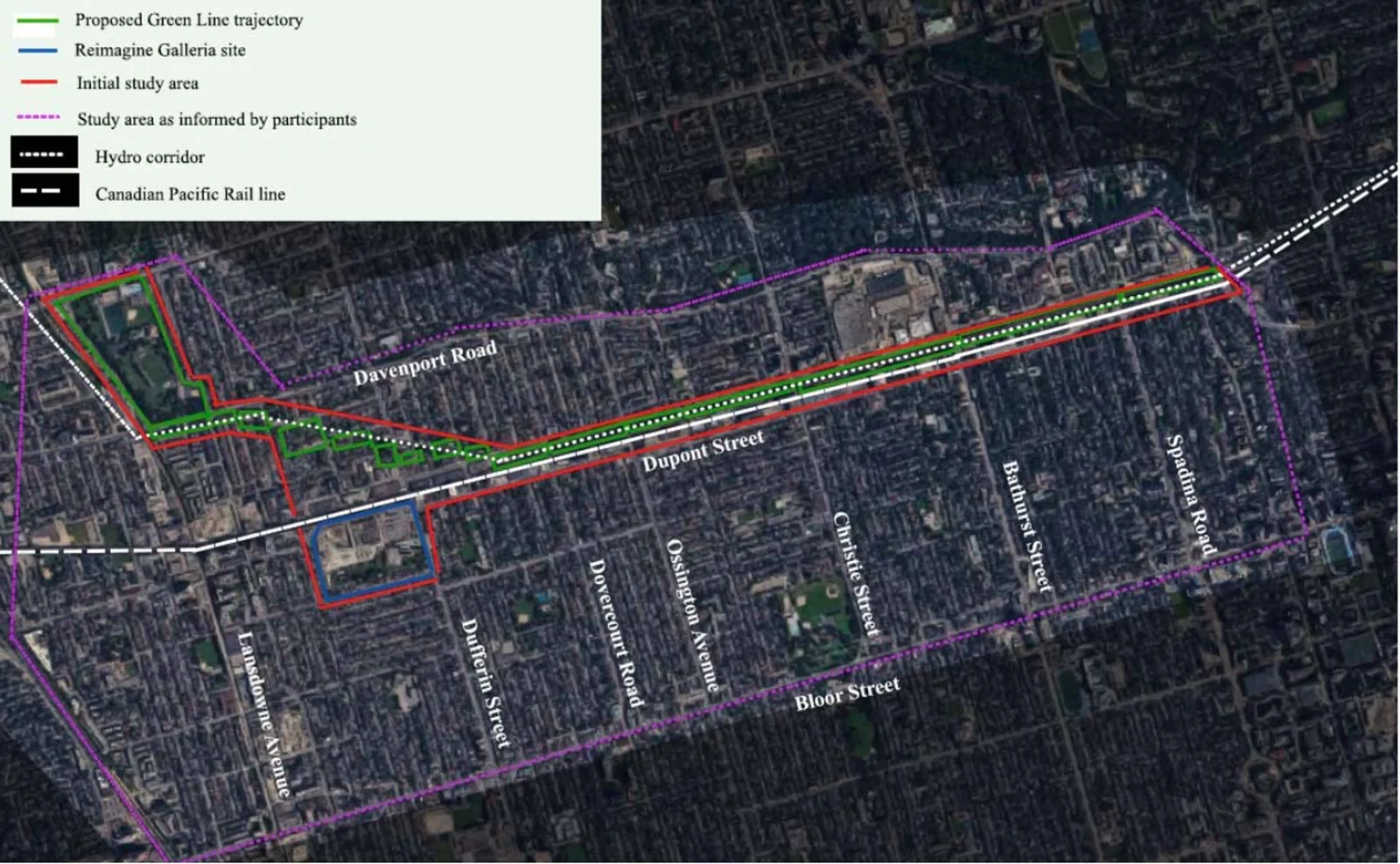
Map of the study area, created by the author.

Studies have shown that while central and eastern parts of the study area have been gentrifying since the 1960s (Walks and Maaranen, 2008; Walks, Hawes and Simone, 2021), the western end began to experience this phenomenon later, in the early 2000s (Kern, 2015; Quinton et al., 2023; Rankin et al., 2016; Rankin and McLean, 2015) alongside efforts to ‘clean up’ the area. Gentrification in the study area takes many forms. Many changes currently underway are led by greening and environmental redevelopment, resonating with the process scholars have called environmental gentrification.

Environmental gentrification – also called ecological or green gentrification – extends the concept of gentrification to explore how natures become entwined in its processes, highlighting how greening and remediation can contribute to exclusion and displacement (Bunce, 2018; Checker, 2011; Dooling, 2009; Gould and Lewis, 2017). As Checker (2011: 212; see also Checker, 2020) points out, environmental gentrification operates in part through ‘a discourse of sustainability’ that renders urban planning practices and development processes politically neutral – if not ‘good’ (see also Angelo, 2021) – both separating sustainability from environmental justice and dissuading resistance to gentrification. Scholars and activists alike have noted the paradoxical character of environmental development and remediation, as discourses of sustainability, health, and wellness have become more prevalent in planning. They urge us to pay attention to how neighbourhood greening – while seemingly inherently desirable – can lead to inequitable outcomes and issues of urban injustice (Anguelovski, 2016; Bunce, 2009, 2018; Checker, 2008, 2011, 2020; Curran and Hamilton, 2012, 2018; Dooling, 2009). Environmental gentrification scholarship draws our attention to vital politics and to biopolitical and necropolitical questions, underlining how health, wellbeing, and futurity become unevenly distributed and to the relations of power that govern, shape, and regulate living (see Parish, 2017). This article explores other ways that environmental gentrification takes place, specifically how it is actively enacted in everyday life, and, furthermore, justified by embodied feelings. While some emerging work seeks to do so (for example Maharawal, 2023), there remains a need to more deeply explore the role of emotion and affect in environmental gentrification processes. I argue that a focus on affective dynamics offers insight into how environmental cleanup and social cleansing intertwine within environmental gentrification, and how activities on the ground are emotionally driven.

Fear has driven organizing and efforts to ‘clean up’ the study area in the past, and contributed to its ongoing gentrification. Fear has been of interest to urban geographers, as an affective phenomenon that shapes places and our experiences of them (Davis, 1999; Harris et al., 2020; Hubbard, 2003; Kern, 2010b; Pain, 2000, 2001; Pain and Townshend, 2002; Valentine, 1989). Fear is political, it exists at multiple scales, it is discursively constructed and deployed at various levels of governance – to justify particular actions or responses, to reinforce divisions, or to bring people together (Shirlow and Pain, 2003). Some geographers studying urban landscapes of fear emphasize how they come together, often involving the production of various ‘Others’ (Pain, 2001; Shirlow and Pain, 2003; Valentine, 1992), and being shaped by a panicked ‘scary city’ rhetoric (Kern, 2010b). Others highlight their relational character, noting that fear plays out in everyday life (Pain and Smith, 2008) and emerges out of everyday experiences of violence (Garmany, 2011). Geographers stress that fear manifests in a variety of ways, often related to perceptions of difference and belonging (England and Simon, 2010), and shaped by factors such as gender, race, and sexuality (Kern, 2010b; Smith, 2010). Discourses and feelings of fear play important roles in shaping cities, justifying the exclusion, control, or policing of anything deviant or disorderly (England and Simon, 2010). In this article, I focus on how Others, seen as deviant, become objects of fear. They become queer and monstrous outsiders, characterized by the ways that they do not fit into local norms, and by the disorder and discomfort they at least *seem* to be the cause of.

This article focuses on the affective roots of trajectories of greening and revitalization that continue in the study area today, further entrenching environmental gentrification. It details ecological practices and grassroots organizing that foreshadowed such initiatives, and shows how stigma and criminalization are emotionally driven processes that have fed into environmental gentrification under the veil of being ‘green’ and thus ‘good’ (Angelo, 2021). The first section of the article details my methodology, focusing on archival analysis as a piece of a broader ethnographic study. The following section provides context for the study, laying out key changes in planning, policy, and governance in Toronto in the 1990s that shape the affective landscape of the city. I then detail how, through the 1990s and early 2000s, fear underpinned ecological and organizing practices in the study area, assembling an affective community of belonging while producing ‘queered’ toxic bodies in the landscape. The section that follows explores how cleanup and greening involve environmental control and forms of policing. Throughout, I show how fear – directed towards a number of seemingly dangerous and invasive more-than-human bodies – serves to justify a combination of policing, redevelopment, and greening as fitting solutions to urban problems.

## Methodology

Affective studies of gentrification are important because affect is a site where its more banal forms of everyday violence play out. Affect is the body's ‘capacity to affect and be affected’, and relates to how bodies are always entangled in transformative relations with one another (Anderson, 2014: 9). Affect is evidential of the body's being and continual becoming in and with the world, and of its ongoing constitution through relations with other bodies. In this work, I pay attention to both affect and emotion: affect is meant to encompass both how bodies are affected by other bodies, and people's ‘interior’ emotional worlds. I am reluctant to divide affect and emotion into two distinct and seemingly bounded fields of research, and prefer instead to think of them as intrinsically entangled (see Anderson, 2014; Smith et al., 2009). I also, however, want to acknowledge important ontological and epistemological differences that exist across some of this work, and the methodological implications that arise as we attempt to study something that is both ‘inordinately diffuse’ and ‘gut-wrenchingly present and personal’ (Smith et al., 2009: 3).

Gentrification, which disrupts one's relational world and sense of place within it, evokes powerful affective and emotional responses. In my research, I have developed a methodology of queer affective ethnography to explore affective experiences and dynamics, infusing affective ethnography with queer thinking. Affective ethnography is a methodological approach theorized and deployed mainly in organizational studies (Gherardi, 2019; Katila et al., 2020; Lubit and Gidley, 2021), but also in more-than-human geographies (Sinha et al., 2021) and science and technology studies (STS) research (Olarte-Sierra, 2019). Scholars underline its attention to a range of elements (including the non-human), to intra-actions and the mutual or contingent constitution of being, and to embodied knowledges, and thus its applicability to the study of complex, entangled, embodied relations between more-than-human actors (Gherardi, 2019; Sinha et al., 2021). Affective ethnography is proposed as a ‘style’ of doing research that is rooted in notions of *being- and becoming-with* (Gherardi, 2019). This implies that research might involve many methods, and that all research is necessarily done *in relation*.

While the critical queering of affective ethnographical work is not by any means a new undertaking, a specifically *queer affective* ethnographic approach has not yet been specifically written about. Authors like Benedicto (2014) and Seitz (2017), for instance, have undertaken queer ethnographies that move beyond the identitarian, both doing queer ethnography and queering ethnography as an approach. I share with these authors an interest in taking queer beyond its use as an identity referent, to speak to how queerness takes shape, and go further in foregrounding the more-than-human. Rooke (2009) suggests that while *queer ethnography* is a useful approach for researching queer lives, *queering ethnography* is different, challenging and reorienting how we do ethnographic research. For many queer scholars, queering means taking both queer theory and queer histories seriously (see Patrick, 2014). For me, queering implies paying attention to discourse, categorization, power, normativity, and how these actively shape webs of meaning. Practically, queering has meant grappling with how normativity operates around affect in the study area, and paying attention to how it shapes who and what belongs. My own approach has involved both examining how queerness as a state of non-normativity is affectively shaped, and tuning in to queer affect: I follow queer scholars who insist on a multitude of registers of affect, think plurally about capacities to affect and be affected, and highlight ‘taboo’ or ‘bad’ feelings in their work (for example Ahmed, 2010; Cvetkovich, 2003; Georgis, 2013; Love, 2007; Martinez, 2014; Muñoz, 2006).

My ethnographic research has involved a multi-methods approach that includes the use of archival research, walking interviews, and immersive observation. This article draws mainly upon archival materials to show how the western end of the study area was constructed in the public discourse through texts and images as a space of fear, danger, and toxicity. I did not set out initially to examine fear. Archival materials rendered visible not only the affective atmosphere of fear in the study area during the 1990s and 2000s, but also provided clarity around how that affective atmosphere was shaped and sustained. My archival practice takes inspiration from queer feminist archivists who have studied and storied the lives of 2SLGBTQ+ communities, challenging what counts as archive and as archival material (see Cvetkovich, 2003). In this work, I have used more formal archival institutions, but I also assembled my own archive of documents, drawing upon newspaper databases, social media websites, community organization websites, and blogs. Through these sources I pieced together glimpses into place, but also gained insight into dominant narratives and discourses that have taken shape related to the study area. Materials were collected between February of 2020 and December of 2022. By the end of this period my archive contained well over a thousand texts, including documents submitted with development applications, policy documents, news articles, social media posts, blog posts, promotional videos, images, pages of feedback from community consultations, dozens of letters to local political representatives, dozens of letters between planners, and dozens of pages of meeting minutes from residents’ associations. Archival research was triangulated with information gained through interviews with long-term residents and people who grew up within the study area.

In analyzing these archival texts, I sought to understand what they do, both individually and collectively. In my analysis I interrogated how belonging and normalcy were constructed and understood in the dominant discourse. I was attuned to the personal and the emotional, and to affective dynamics playing out through and between the texts. I looked for expressions of emotion and affect, paying attention to what specific human and non-human entities these related to. In written documents, I noticed ways of characterizing and talking about people, places, and things. In images, I noticed subjects of focus, and different ways of representing particular landscapes and bodies. I identified fear and anxiety as key affects circulating in and among these documents, and as forces consistently connected to and driving greening, redevelopment, and gentrification.

## Toronto's west end in the 1990s

Understanding the larger policy, planning, and development contexts of Toronto in the 1990s and early 2000s helps to better contextualize the affective space of the study area during this period of the city's history. This broader context also complicates the narrative propagated by the media about the study area being a ‘family community’ (Van de Ven, 2003, n.p.) under attack by fearsome invasive forces, instead revealing broader political and economic processes at work. Emerging place-specific anxieties existed against a backdrop of political and economic restructuring in Toronto, and amidst efforts to intensify development in the central city. We can see how particular revanchist solutions were deployed in response to these anxieties.

Urban neoliberalism, and the accompanying shift away from Fordism and the state provision of social services that had previously helped solidify Toronto's reputation as the ‘city that works’ (Donald, 2002), led to increased socio-spatial polarization in the city. Reinvestment and redevelopment transformed downtown neighbourhoods into landscapes of affluence, while poor, low-income, and working-class residents became increasingly concentrated in particular inner-city and inner-suburban neighbourhoods (MacDonnell et al., 2002; Walks, 2001). The 1990s were, as Kipfer and Keil have described them, ‘hard times’ (2002: 229) for anyone living on the city's fraying social edges. During this decade, the city was affected by both economic recession and the election of the new Progressive Conservative government of Mike Harris at the provincial level in 1995. Promising a ‘Common Sense Revolution’, Harris’ government began a program of reform, cutting programs and government spending, defunding major infrastructural projects, laying off government workers, and downloading key services to the municipal level.

These moves have been linked to increasing inequalities (Joy and Vogel, 2015), deepening poverty (see MacDonnell et al., 2002), and a long-term increase in homelessness in Toronto (Greene, 2014). Additionally, policies were created that were punitive to the most poor and marginalized people in society. Examples include the *Safe Streets Act* (1999) which aimed to eliminate the public presence of stigmatized ‘pan-handlers’ and ‘squeegee kids’ (see Hermer, 2019; Hermer and Mosher, 2002; Keil, 2002) and prohibited the public disposal of objects like used condoms and needles, and the Community Action Policing (CAP) program which sought to target ‘criminal hotspot’ neighbourhoods where unhoused people dwelled (Greene, 2014: 28). Following the provincial government's forced amalgamation of Ontario municipalities in 1998 (which amalgamated the four cities and two boroughs that made up the area known as Metropolitan Toronto into one central government) the crisis of poverty in the central city ‘deepened’ (Greene, 2014: 28).

Municipal governance, meanwhile, turned towards the promotion of Toronto as a global city. Under the direction of newly elected mayor Mel Lastman, Toronto began to employ more entrepreneurial approaches to governance and planning, seeking to increase the city's competitiveness through the development of new cultural infrastructure and megaprojects. The city also displayed characteristics of revanchism, entrenching race and class divides while controlling and policing downtown spaces to make them ‘safe, clean, and secure for investors, real-estate capital, and the new urban middle classes’ (Kipfer and Keil, 2002: 237; see also Greene, 2014). The shift from social policy to crime control involved an aggressive new ‘law-and-order agenda’, underpinned by Wilson and Kelling's (1982) ‘broken windows’ theories about neighbourhood policing that suggest that the presence of visible signs of disorder will lead to the decline of a neighbourhood (Kipfer and Keil, 2002; see also Hermer, 2019; Hermer and Mosher, 2002). These new practices buttressed place-specific processes of ‘reurbanization’ in Toronto, as a wave of condominium development and revitalization initiatives began to transform the inner city (see Desfor and Laidley, 2011; Kern, 2010a, 2010b; see also Lehrer, Keil and Kipfer, 2010).

Understanding this broader context helps to reveal compounding and layered processes transforming the study area during this time. This context is important when considering changes occurring and what kinds of stories are told about those changes and their visible, place-specific effects. The context in which poverty has increased in the city is noticeably left out of narratives surrounding the study area and its cleanup, and in its stead we can observe a discourse of contamination and sanitation.

## ‘Cleaning up’ the neighbourhood

My archival documents show how, starting in the 1980s and increasing into the mid-2000s (as gentrification begins), associations with dirt, pollution, and immorality began to contribute to the study area's construction as a kind of wasteland. Textual records in news coverage, blogs, emails, and message boards present certain bodies as pure and belonging, and others as dangerous invading forces that must be removed. Their discourse reflects fears and anxiety around degradation that are not just about environmental purity but also about social purity. Societal fear about urban disorder, dirt, and decay are often related to fears about what Brownlow calls a city's ‘weedy ecologies’ (Brownlow, 2006). As can be observed throughout my archival texts, leakages occur between human and non-human as ‘abject subjects’ (Chen, 2012: 40) are produced. Bodies swirl together in an assemblage wherein anxiety and fear are binding agents.

Media plays a central role in storying the study area and shaping this affective atmosphere, drawing attention to particular locations, bodies, and events. Articles ensnare specific spaces in a discourse of ugliness and danger that has justified past and current planning and development imperatives to improve, redevelop, and green. For decades into the mid-20th century, vast swaths of the study area were locations of large-scale industrial production surrounded by working-class neighbourhoods. The toxic remnants of these uses are very real, and measurable levels of contamination continue to exist across parts of the area today. These are testaments to the area's longstanding industrial character, with smaller scale industry maintaining an ongoing presence. Local streets like Geary Avenue and Dupont Street are well known for this. This landscape of factories, mechanic and repair shops, warehouses, and parking lots has in the past – before ‘grit’ had any cultural cache – seemed hostile and uninhabitable to many non-residents, described as a ‘no man's land’ and as ‘cut off from Toronto's urbanized core both physically and spiritually’ (Berman, 2015, n.p.). Through the 1990s, these locations were framed as spaces of fear.

In an article titled ‘Track Marks’, published in the bourgeois magazine *Toronto Life* in 2004, Andrea Curtis contrasts the ‘busy, energetic’ multicultural family life of the daytime with the ‘sinister’ atmosphere of danger at night. Curtis offers up an array of characters who are represented as distinctly out of place: ‘A jittery 20-year-old in a red tuque and shiny brown jacket strides purposefully, looking over his shoulder every few paces. A girl in a tight tiger-print skirt and knee-high boots stands just off the main drag, near the old movie theatre turned dollar store, her pose simultaneously defensive and expectant’ (2004: 113). Curtis identifies particular spaces and bodies as deviant. Parts of the built environment, such as low-income housing, unoccupied buildings, and ‘third spaces’ like donut shops, restaurants, or ‘dive’ bars occupied by lower income groups, come to represent a place that is seemingly coming apart. Vacated buildings are described as overrun by stray cats or drug users (the lines between different forms of undesirable ‘vermin’ frequently blur). Parks, stairwells, and doorways are imagined as sites of illicit activity such as drug consumption, the sale of substances, or sex for money. Alleyways and passages are described as overgrown, while bushes and weeds provide cover for crime. In the media specific streets are constructed as locales of risk and danger.

These narratives are echoed in how people affectively experienced the spaces. Recalling this period, one research participant, Diane, who spent her childhood growing up in the area near Wallace Emerson Park, recounts being told that local streets were ‘notorious for drugs and prostitution’ (Interview September 28, 2021) and that they were unsafe. Two other participants who grew up in the same area recall understanding that their neighbourhood was dangerous, but also recognizing that it was heavily stigmatized (Interview December 2, 2021). One, who grew up in subsidized housing provided through Toronto Community Housing Corporation (TCHC), recalls that the community during the 1990s was insular and made up largely of lower-income, racialized immigrant families, many of which were single-parent, female-headed households. She recalls that her housing was ‘inward-looking’ in its built form, that it was viewed from outside as ‘downtrodden’, and that it was derogatorily characterized by people who did not live there as ‘the projects’ (Interview December 2, 2021). These participants explain that they knew they were from the ‘wrong side of the tracks’, but they now knew that this perception of the neighbourhood was related to socio-economic factors like class, race, and immigration status that they did not totally comprehend as children.

Into the early 2000s, news articles discussing the west side of the study area describe it as a place of crime and decay. In 1993, Ward 11's City Councilor, Rob Maxwell, notes that media coverage during this time did ‘more harm than good’ (in Small, 1993, n.p.), giving the area a bad image. We can see this discourse as being part of a broader ‘scary city’ discourse that emerged in Toronto and arguably climaxed in 2005 during the ‘summer of the gun’^1^ (see Kern, 2010b). In broader terms, danger is tied to numerous incidents of violent crime in the area, which largely the result of economic deprivation and pockets of concentrated poverty, and were major focal points of media attention in Toronto through this period. Throughout the 1990s and early 2000s, the majority of media articles discussing the western end of the study area focus on and often sensationalize various local forms of disorder. Amidst evidence of long-standing neighbourhood disinvestment and increasing local poverty (see City of Toronto, 1974; Lyon and Newman, 1986; McLean, 2014), a constructed externalized criminal element takes most of the blame for the deterioration of this part of the study area. Homeowners in the area are frequently framed as being in a pitched battle or ‘war’ (McKnight, 2014, n.p.), or on a ‘crusade’ (Small, 1993, A4) against crime. ‘Pushers and prostitutes’ and ‘blight’ (Small, 1993, n.p.) are blamed for ruining what was once a pleasant and family-oriented immigrant neighbourhood.

## More-than-human affective assemblages: Needles, sex workers, and at-risk youth

Examining my archival documents, I observe that a sense of deviance and danger sticks to identified bodies, who are understood within the dominant narrative as not belonging, being instead invasive and ‘out of place’ (also see Kern, 2015). This resonates with observations made in the work of Kern (2015) and Dillon (2014) on place-specific relations of toxicity, the production of waste-able spaces and bodies, and the justification of clean-up. Undesirability, danger, and toxicity are tied to the presence of often racialized and lower-income or poor people who are repeatedly framed as ‘outsiders’, and to the presence of a range of criminalized informal economies involving drugs or sex work that emerged locally as marginalized people struggled to survive in the city. As such, imperatives to ‘clean up’ the neighbourhood are not only concerned with ‘toxic’ environmental factors like contaminated industrial sites scattered throughout the study area, but also with a wider array of marginalized bodies that represent threatening disorder. Toxicity spreads across bodies, both human and not, as they become identified as matters of concern and discursively connected. Affect is a kind of lubricant enabling this connective process. Environmental cleansing becomes entwined with social cleansing as these bodies become enmeshed.

My archival analysis identifies an increased ‘public concern’ about drugs and visible drug use starting in the 1990s. The syringe, for instance, is a non-human object that wields enormous power in this landscape, invoked to justify a range of interventions – most notably policing and securitization. Archival materials such as residents’ association message board posts and interviews with residents in the press reveal a perception of and focus on it as a fearsome entity (see Figure 2). Symbolically and materially, it is connected to the drug trade and to drug use. In one newspaper article, a local resident explains: ‘(T)he drug problem has become more visible around here in the past 2 years [1989–1991]. Crack houses are popping up, hookers who were chased out of other areas are showing up around here and we’re finding dirty needles in the park next to our clubhouse. Not a great environment for kids’ (in Sokol, 1991, n.p.). The focus on children's safety underpins the urgency of the issue, driving calls by parents and non-parents alike for immediate intervention on the behalf of a population who are framed as pure and innocent. In another article, a local beat police officer describes sex workers using park spaces for business, saying: ‘(T)here's needles, condoms in the same place where kids are playing soccer’ (in DIG IN, 2006, n.p.). The needles, it is argued, ‘could be infected with AIDS’ (in Swainson, 1988, A1) and make park spaces unusable and dangerous, particularly for children.

**Figure 2.**
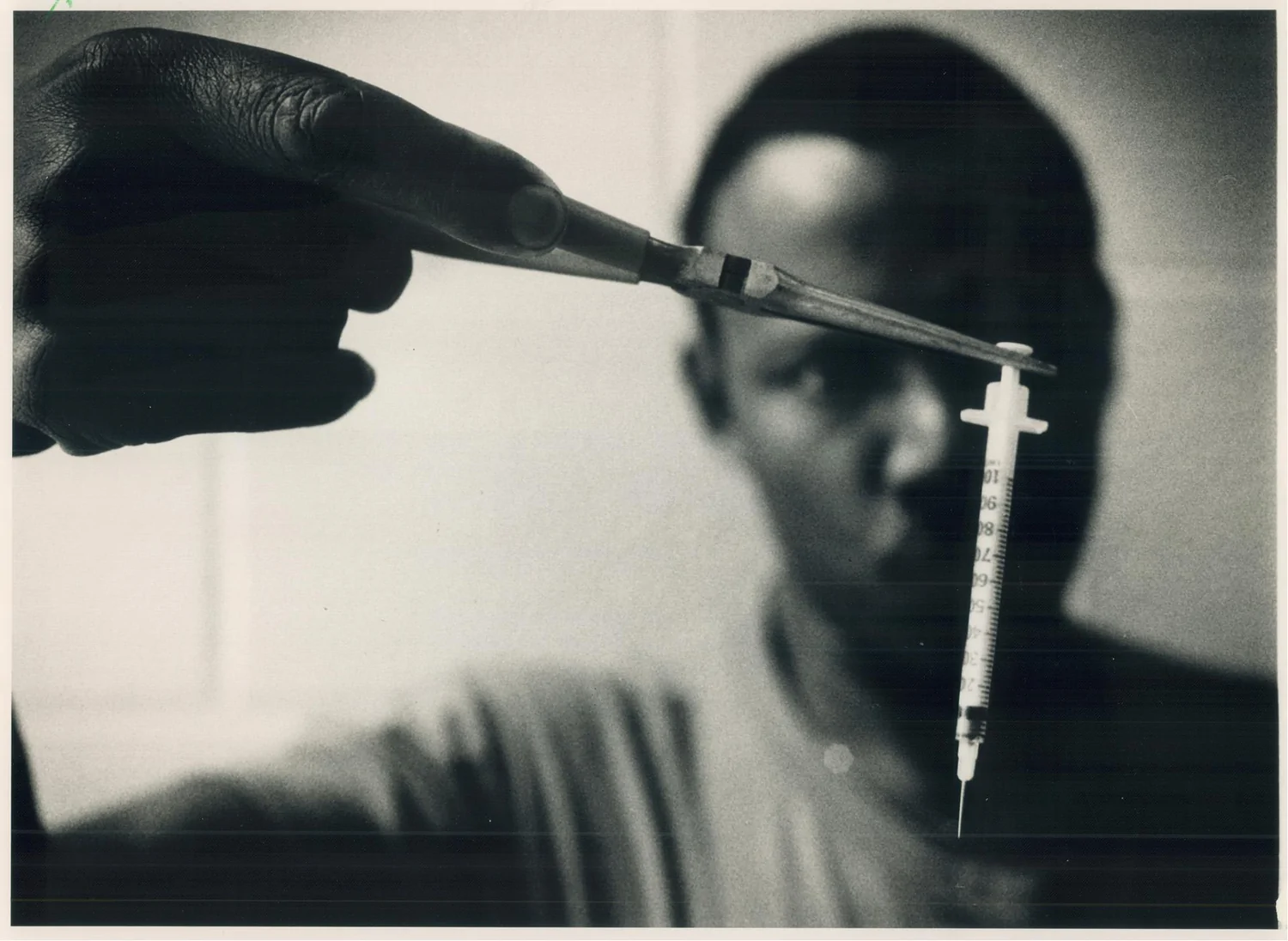
Image of a Bloordale community worker holding a found syringe. Photo by Boris Spremo, 1988 (Toronto Star).

The discarded hypodermic needle in a park is a well-known local symbol of disorder that is encountered with a particular horror, perceived to be heralding the invasion of drug users into the neighbourhood and terrorizing local residents (in particular parents of young children). The needle, used and cast aside, is often viewed as an object of fear and deployed to justify the surveillance of public space and the removal from these spaces of a range of seemingly dangerous people. In the archive, the object of the syringe becomes entangled with and comes to represent undesirable human populations, serving to render drug users (as well as many people who may not actually use drugs) undesirable, dangerous, and ultimately, like the needle itself, disposable. The object is symbolic of what one *Toronto Sun* article terms a ‘junkie invasion’ (Kingstone in DIG IN, 2006, n.p.) and is used as a factor to justify the removal of people who were perceived as unruly outsiders. In one article, local anti-drugs organizer Dennis Fatino blurs boundaries between social and environmental cleansing, stating: ‘The immediate problem is drugs. Let's deal with that first and then we can deal with the garbage problem. We have a drug problem. We have a garbage problem, but *it's a human garbage problem*’ (in Swainson, 1988, A1, emphasis mine). In archival texts, when local homeowners and parents highlight the presence of needles in local parks at community consultations and public meetings, they also implicate the people who have become enmeshed with these needles, and they argue for their policing and removal from the space. The presence of needles, to them, signifies the increasing ‘boldness’ of dealers in the community and drives the desire in some homeowners to take their neighbourhoods back by force (Swainson, 1988, A1). As one article states: ‘After years of watching the junkies take over their streets, residents… are saying they have had enough. No more junkies shooting up in broad daylight behind the schools, no more needle-strewn parks, no more addicts sleeping on their front lawns’ (Swainson, 1988, A1).

Rather than sparking the creation of safe injection sites and needle exchanges, the used syringe is instead deployed to discourage the development of homeless shelters, to push back against low-income housing, to shut down the location of harm reduction service centres, and even as justification for outright confrontation with drug dealers and drug users in the streets. In the late 1980s, residents organized anti-drug use marches with the support of the police, and organized ‘rallies featuring face-to-face confrontations with the dealers’ who were identified through a list of out-of-area license plate numbers collected by organizers (Swainson, 1988, A1). As organizer Fatino explained: ‘It's a matter of harassing them’ (Swainson, 1988, A1). These practices are framed as the ‘community taking a stand’ in order to ‘get our parks back and our streets back’ from ‘drugged-out urban fringe-dwellers who have come perilously close to making the streets their very own’ (Swainson, 1988, A1). Archival documents such as residents’ association internal emails and posts on public message boards show that many of these policing efforts resulted in large numbers of arrests. Cleaning up the neighbourhood becomes about more than the removal of needles from public space. It is also oriented around the removal of undesirable people, with fear of dirt, toxicity, and contagion justifying the policing of perceived drug users and dealers.

Similarly, the body of the sex worker is constructed within my archive as a matter of concern. This body is queerly and precariously put together, made up of both human and non-human elements; its non-belonging is shaped by affectively charged presuppositions about the materialities and embodied practices that make it up. In other words, the sex worker is a kind of monstrous entanglement, a patchwork figure of deviance who comes together in the archive as an overly sexualized, more-than-human assemblage. The sex worker can be found throughout the neighbourhood – lurking in the short skirts and defiant looks of random women on the street, detectable in anyone standing outside the two strip clubs located along Bloor Street. Anxiety attaches to a range of non-human objects that might signal the sex worker's presence in the environment – a high boot, a miniskirt, a used condom, the aforementioned syringe. Drug use and sex work go hand in hand in local narratives about sex workers. Using features high on the list of their many possible unruly behaviours – in some of the media coverage and in the language of some local residents and community organizers, sex workers are repeatedly referred to derogatorily as ‘crack whores’ (Balzer, 2007; Hutcheson, 2006; see also McLean, 2017).

Sex workers are framed in archival documents as part of a new wave of decay in the neighbourhood; a sign that things are getting worse and that residents are living ‘in a different world’ (Sokol, 1991, n.p.) than they once did. They are often framed as an infestation, with one resident of the study area claiming in the media: ‘There are times you can literally not walk on the streets because of the hookers’ (in Swainson, 1988, A1). Sex workers are claimed to produce an ‘edgier’ atmosphere, apparently drawing drinkers, pimps, and drug dealers into the area (in Lukashevsky, 2021: 19) while also being drawn by drugs that can be found locally (in Swainson, 1988). Newspaper articles discussing this invoke better days, a yesteryear of postwar prosperity (Small, 1993) that is contrasted with an emergent and seemingly anarchic, drug-fueled, and hypersexual present.

The sex worker is often contrasted against the nuclear family. Their presence is understood by many residents who are organized around cleaning up the neighbourhood to be dangerous – especially to children – and one that, as with the syringe, must be eliminated. In one article, a long-time resident states: ‘I grew up in an Italian family with eight kids. Sure, we had neighbourhood drunks, but there were no drugs, no hookers, and no swarming’ (Small, 1993, A4). The resident goes on to suggest that the presence of these out-of-place bodies and practices is both cause and symptom of the breakdown of morals, established traditions, and family life. Fear of the sex worker, as a queered figure exuding uncontrolled sexuality and moral deprivation, is, of course, not unique to this place or this time. Sex work has long been considered ‘the greatest social evil’ in cities, as a kind of ‘moral miasma’ with ‘the potential to destroy the family, shake the foundations of society, and spread disease’ (Kern, 2019: 9; see also Hubbard, 1998; Hubbard and Sanders, 2003; McKenna, 2022). Here, there is a sense that the perceived immorality of the sex worker is contagious, that their very presence will further spread the disorder that they represent.

Finally, in the body of the ‘at-risk youth’ exists another unruly and disruptive force of discontent. The ‘at-risk youth’ denotes an amorphous assemblage of bodies, an uncontainable multitude capable of many things. In archival documents, local residents start to express worry about young people, observing increased drug use among them, as well as an increased ‘disrespect for authority’ (in Sokol, 1991, n.p.). Residents quoted in the media express concerns about youth being drawn into gangs and lured into the drug trade. This story typically features neighbourhood youth being influenced by sinister and criminal forces originating outside of the neighbourhood. Again, the discourse suggests an invasive presence is degrading the ‘purity’ of the neighbourhood, that is once more embodied in the figure of innocent youth. The youth becomes ‘at-risk’ as they are exposed to this malignant outside force, potentially losing their status of belonging to the neighbourhood as they are corrupted. The normative discourse in local media suggests that these youth stray from normative family life, becoming anti-social and careless of how they affect others.

The individual young person is constructed as vulnerable (‘at-risk’) and as a potential target, but as a group the bodies of ‘at-risk’ youth become a roving and unpredictable animalistic pack – a matter of concern for residents and police. As an amorphous pack, the youth become dangerous, unpredictable, and threatening. Groups of youth are described as hanging around public spaces such as neighbourhood playgrounds and skate parks, existing as a ‘swarm’ (in Small, 1993, A4), causing ‘disruption’, and contributing to an ‘emotional roller coaster’ with their ‘loitering, obscenities, ominous mood’ (in Steed, 1994, n.p.). Boredom, alienation, and lack of motivation are seen as a recipe for disaster, causing kids to skip or drop out of school and to be drawn into these groups (Steed, 1994)*.* Many of the community initiatives directed towards improving the neighbourhood focus on taming these bodies and bringing them under control through a variety of programs, reintegrating them into traditional family life and teaching them proper values.

In all these narratives, heteronormative, middle-class, nuclear family life is often set up in opposition to queered forms of degeneracy or excess. The media narratives in my archive produce the bodies of low-income people, racialized people, young people, drug dealers, drug users, sex workers, neurodiverse people, people with mental illness, and mental health survivors who inhabit the area as outsiders threatening a desired socio-spatial order. As local community relations officer Art Dean stated in 1993, more than 90 per cent of people being arrested in the area at that time were ‘outsiders’ (in Small, 1993, A4). Through the 1990s, ‘outsider’ reads as someone who simply does not live in the neighbourhood; someone who is not a member of the constructed community of respectable, working families who are repeatedly affirmed as the rightful inhabitants of the study area; and, ultimately, someone who is potentially dangerous to the neighbourhood. These outsiders are framed as invasively out of control. One article notes that arrests only provide a short-term solution to the removal of disorder, as droves of criminals simply ‘backfill’ (Osiewicz and Sarick, 1999, A10). The neighbourhood is imagined in the media as a kind of pure, unified, vulnerable, and threatened entity that must continually fight off a threat. Living in the neighbourhood is framed as a terrifying, frustrating, and tearful ‘battle’ (Small, 1993, A4) against an ever-oncoming force.

In highlighting the discursive construction of certain bodies as toxic and invasive I do not wish to ignore the very real violence that marked the period between the 1980s and the early 2000s in the study area. Media articles both contribute to and describe the study area's affective atmosphere of anxiety and fear. Through interviews with long-term residents and people who grew up in the area, it became clear that locals’ everyday affective experiences were shaped by frightening things happening in the world around them. Repeated, everyday incidents of violence powerfully shaped the affective worlds of residents. These are also documented in the archive: one news article spotlighted the stabbing of a local rooming house resident (*Globe and Mail*, 1990), another speculates about the origins of a sawed-off semi-automatic rifle found in a laneway (*Globe and Mail*, 1992). Tales of shootings, stabbings, and bludgeonings abound. These horrific events pile up, but in the media they are never connected to broader contexts of disinvestment or of social, political, and economic restructuring that they occur within. This disconnect is reflected in the perceptions of residents, and maintains disorder as somehow inherent to queered bodies, rather than produced by broader social factors.

One journalist, Curtis (2004), explains how the western end of the study area became ‘clearly and horrifically marked’ (110) as a troubled neighbourhood through the 1990s through experiences that were collectively traumatizing. She details the raw emotional state of the community as ‘hysteria reached its zenith’ (110) in 2002, following the murder of Holly Jones. Jones was a local girl who was kidnapped in broad daylight after walking a friend home from Sterling Road to Perth Avenue, on the same route she took to and from school every weekday. Curtis describes the atmosphere of vigilance that emerges through these kinds of incidents – ‘watchful parents gripped their children's hands as they walked them to school. Parks lay vacant. Police held a crash course in streetproofing, and residents called on [then Toronto Police Chief] Chief [Julian] Fantino… to send more police cars and foot patrols into the area. Even deep into last summer, the dead-end streets that back onto the railway tracks – the site of fiercely contested street hockey games – were unusually quiet’ (2004: 113–114). For weeks after the incident, until Jones’ murderer was arrested, residents feared a ‘monster’ (Brown, 2003, n.p.) was loose in the area.

In news articles, there is a sense of a community coming into being through shared suffering, grief, fear, and vigilance. In particular, this seems true for families with children, who, as one resident puts it, ‘all felt that it could have been our child. We formed a bond over it’ (in Curtis, 2004: 113). As another resident explains: ‘In some strange way, the murder, horrible as it was, created a common experience. There was a certain kind of jelling around it. Not focused on revenge so much as cleaning up our neighbourhood, getting to know our neighbours’ (in Curtis, 2004: 114). Articles suggest a togetherness produced by a sense of collective victimization. Many residents, elected representatives, and local business owners describe an atmosphere of fear and a sense of being ‘fed up’ (Small, 1993, A4). This drove local residents to start organizing, attending community meetings, working with local police forces, and engaging in environmentally-focused practices like trimming back vegetation and improving lighting in the public realm in order to dispel ‘pushers’ who were ‘prey[ing] upon’ the neighbourhood (in Small, 1993, A4). Curtis’ article concludes that ‘(M)aybe something good came out of something very bad’ (2004: 114). Here, a community emerges out of shared trauma, directing itself towards the task of ridding itself of objects of fear.

The characterization of more-than-human bodies in the landscape as invasive and the entanglement of some bodies with danger, toxicity, or waste produce a drive to ‘clean up the neighbourhood’, and to return it to a rightful community of families and homeowners. Affective experiences of fear produce a shared sense of urgency, driving both community-led and policy imperatives to ‘purify’ this residential neighbourhood from what is viewed by vocal homeowners, residents’ associations, and the media to be a toxic and invasive culture of drugs, crime, and disorder. As a pragmatic approach for addressing social problems, greening becomes a way for the community to deter invasive forces, remove bodies that don’t belong, and restore itself to its seemingly natural balance as a peaceful, family-friendly neighbourhood. Here, greening is taken for granted as a positive activity. No connections are made between it and possible forms of environmental injustice.

## ‘Green, clean, safe, and civil’

Fear assembles an affective community and drives both environmental and social cleansing efforts, justifying policing, redevelopment, and eventual trajectories of greening as solutions of perceived urban problems. Dovetailed goals of environmental and social ‘cleanliness’ blur lines between greening and policing. Archival documents show that within the study area, activities start to be undertaken that align with the ideas laid out in *Crime Prevention Through Environmental Design* (CPTED). This is an approach of the Toronto Police Services (TPS) popularized in the 1990s (and still applied today) which follows the logic that crime decreases if a property is a ‘less appealing target’ (Toronto Police Services, 2002). The idea here is that disorder can be managed or even prevented through environmental strategies. CPTED is informed by broken windows theory. As broken windows theory argues: ‘when a property does not look like it is cared for, it communicates to potential offenders that there is no one watching over the property, so there is no one to protect it’ (Fennelly and Perry, 2018: 284). Preventing crime, in this logic, involves creating opportunities for surveillance, controlling access to and movement through space, and reinforcing the sense of community ownership over certain spaces. Residents seeking to reclaim their neighbourhoods must ‘demonstrate territoriality’ (Fennelly and Perry, 2018: 284). This involves forms of citizen-based policing, as well as controlling and maintaining the more-than-human environment – in other words ensuring that properties are well-lit and maintained, lawns are mowed, properties are clearly demarcated, and that elements like hedges or flowerbeds are strategically placed.

In the study area, environmental control involves particular practices and ecological relations. Foliage in back lanes is trimmed back to enable surveillance, while the planting of trees is understood as a beautification practice capable of deterring crime. In earnest, residents plan cleanups in parks. Letters are written to city councilors demanding environmental issues such as weeds or garbage in the streets be addressed, complaining about waste items being dumped in various sites, and pushing for beautification measures in public spaces. Reports published through the 1990s by ratepayer groups like the Bloor-Lansdowne and Bloor-Ossington Committees Against Drugs recommend a range of physical and economic revitalization schemes for the western end of the study area. These groups and their reports express hope that redeveloping commercial and leisure landscapes will reduce perceived social issues and restore the neighbourhood to its ‘rightful’ communities. During this period, community groups meet extensively with police to discuss environmental solutions to crime, such as the installation of lighting, graffiti removal, and the creation of ‘neighbourhood watch’ groups to surveil public spaces.

Amidst this flurry of emotion-driven activity we can observe the coming together of local organizations such as the Dupont Improvement Group: Improving the Neighbourhood (better known as DIG IN), which was mainly active from 2004 until the mid-2010s, as gentrification became entrenched. DIG IN was dedicated to making neighbourhoods at the western end of the study area ‘green, clean, safe and civil’ (DIG IN, 2015, n.p.). The group's efforts to claim space dovetail with efforts to police the area, as DIG IN established an ongoing collaborative relationship with TPS. The coalition of actors that come together to form DIG IN pursue an ‘environmental approach’ meant to ‘ensure places for families, artists, diversified commerce, green spaces’ (DIG IN, 2015, n.p.). Through the early 2000s, they push for community regeneration oriented around a combination of artistic interventions and greening, promoting a ‘progressive’ approach (DIG IN, 2005, n.p.). Amidst the group's efforts to have the area adopted by the city as an ‘economic revitalization zone’, art and greening become deeply intertwined as tools able to ‘revitalize, green and connect’ an ‘at-risk community’, and address the ‘depressed atmosphere arising from its urban troubles’ (DIG IN, 2005, n.p).

The work of DIG IN has already been critiqued, but not examined in terms of its role in environmental gentrification or its more-than-human or affective politics. Heather McLean (2014, 2017) offers a critical perspective on DIG IN's activities, revealing the contradictions and tensions inherent in their organizing during this period, as well as their role in claiming space for homeowners, middle-class gentrifiers, and affluent visitors to the neighbourhood. As McLean explains, ‘even though Dig In's activities ostensibly promoted alternative community development and supported queer and feminist arts organizations, the group also naturalized the production of depoliticised spaces of middle-class feminism, homonormativity, and white privilege’ (2017: 39). In my own research I observe similar dynamics around greening efforts and community-led cleanup initiatives, as well as connections between these initiatives and arts-led revitalization. I see that environmental gentrification involves the categorization and identification of undesirable others by those with power, as well as a marked effort to remove them from spaces meant to be appropriated by an apparently more deserving community. Furthermore, the two processes are entwined. In both, displacement and replacement are understood and undertaken as a supposedly ‘good’ acts, framed in terms of improving the neighbourhood, ‘reclaiming’ it, and restoring it to a ‘healthy’ state. Yet, cleaning up the neighbourhood can be seen ultimately as a form of revanchism draped in a discourse of sanitation and improvement, made appealing to a liberal middle class who, by engaging with art or greening and by ‘reclaiming’ space, become more personally implicated in gentrification processes. This binding of appropriation and displacement to notions of health, cleanliness, and improvement allows both established homeowners and newly arrived gentrifiers to have good feelings about participating in these processes. Meanwhile, the project of making the neighbourhood ‘green, clean, safe, and civil’ involves the policing, removal, and funneling of unwanted and criminalized ‘outsiders’ into such systems, and their replacement with more ‘desirable’ individuals who are never themselves framed as ‘invasive’.

## Fear, disorder, and displacement

‘Outsiders’ take shape through affective dynamics. Within the study area, fear has been a collective affect, circulating in a feedback loop between residents and the media. Affect saturates the landscape and is *sticky* (Ahmed, 2010); fear looms and attaches easily to bodies. Queered bodies – construed as invasive and potentially dangerous – have stood out in how they trigger fear, embody local anxieties, and inhabit categories of disorder and invasiveness. This has very real detrimental effects: as assemblages of fear have taken shape, we see more-than-human communities made vulnerable to harm and displacement, while privileged residents engaged in ‘cleanup’ practices perceive themselves to be participating in something ‘good’. Examination of these affective dynamics reminds us that gentrification is both systemic and actively enacted in the everyday, its politics and processes charged with feeling. Seen through the prism of affect, these efforts emerge out of and are shaped by an atmosphere of anxiety and fear. This symphony of vernacular ‘cleaning’ and ‘greening’ practices constitute the everyday efforts of the community's seemingly more legitimate members to brave their fears, reclaim the neighbourhood from seemingly out-of-place invaders, and exert control over existing local ecologies. These practices have served to police, uproot, and displace ‘weedy’, more-than-human ecologies of marginalized people and unmanaged ecosystems that have taken root.

Over time, residents’ organizing efforts have reshaped the landscape, setting the stage for larger-scale ‘green’ redevelopment initiatives we see today, such as Galleria on the Park or the Green Line. A current surge of parks-led redevelopment and environmental gentrification in the study area follows on the tails of this previous era of neighbourhood cleanup, involving moves to remediate and ultimately re-work more-than-human worlds for the benefit of a new wave of gentrifiers. This follows the arguments of environmental gentrification scholars who suggest that remediation and redevelopment tend not to serve the undervalued residents who have had to live in marginal urban spaces, been exposed to industrial toxins, and suffer their effects (see for instance Anguelovski, Connolly and Brand, 2018; Gamper-Rabindran and Timmins, 2011). The media and planning discourse surrounding projects like Galleria on the Park and the Green Line frames the greening of formerly industrial space as both ‘good’ and as part of a ‘natural’ process of neighbourhood change, giving shape to new constructed outsiders while naturalizing and depoliticizing their displacement and replacement. Gentrification and displacement are framed as inevitable. This affectively fueled construction of outsiders occurs alongside the active construction of communities of belonging.

An affective examination of environmental cleanup and redevelopment, as well as the everyday practices that these involve, reveals the intense feelings that produce ‘toxicity’ and underpin exclusion and displacement, and that drive greening practices. This adds nuance to our understandings and accounts of how environmental gentrification takes place. By understanding how bodies are affectively invested with meaning, we can better see how risk is unevenly distributed, and how both belonging and non-belonging take shape in particular places. We can more fully understand not only how displacement proceeds, but how certain beings are marginalized and readily marked for intentional removal, and further, how their removal becomes justified and even potentially understood as a positive outcome.

## Notes

1. This name was bestowed upon the summer of 2005 by National Post correspondent Natalie Alcoba, who wrote of gun violence and the 33 shooting incidents that had occurred between June and August of that year.
